# Spin-Glass Transitions in Zn_1-x_Fe_x_O Nanoparticles

**DOI:** 10.3390/ma13040869

**Published:** 2020-02-14

**Authors:** Lilian Felipe S. Tupan, Marlon I. Valerio-Cuadros, Aline Alves Oliveira, Reginaldo Barco, Flávio Francisco Ivashita, Lutiene F. Lopes, Edson C. Passamani, Andrea Paesano

**Affiliations:** 1 Departamento de Física, Maringá State University, Av. Colombo, 5790–Zona 7, Maringá, PR 87020-900, Brazil; lilian.tupan@hotmail.com (L.F.S.T.); marlon190@gmail.com (M.I.V.-C.); alineuem@hotmail.com (A.A.O.); r_barco@hotmail.com (R.B.); fivashita@gmail.com (F.F.I.); 2 Instituto de Física, Federal University of Rio Grande do Sul, Porto Alegre, RS 91501-970, Brazil; lutienefl@gmail.com; 3 Departamento de Física, Federal University of Espírito Santo, Vitória, ES 29075-010, Brazil; edson@cce.ufes.br

**Keywords:** Fe-doped ZnO, dilute magnetic semiconductor, spin-glass, energy bands, X-ray diffraction, diffused reflectance spectroscopy, magnetic properties, Mössbauer spectroscopy

## Abstract

Monophasic Zn_1-x_Fe_x_O nanoparticles with wurtzite structure were synthesized in the 0 ≤ x ≤ 0.05 concentration range using a freeze-drying process followed by heat treatment. The samples were characterized regarding their optical, structural, and magnetic properties. The analyses revealed that iron doping of the ZnO matrix induces morphological changes in the crystallites. Iron is substitutional for zinc, trivalent and distributed in the wurtzite lattice in two groups: isolated iron atoms and iron atoms with one or more neighboring iron atoms. It was also shown that the energy band gap decreases with a higher doping level. The samples are paramagnetic at room temperature, but they undergo a spin-glass transition when the temperature drops below 75 K. The magnetic frustration is attributed to the competition of magnetic interactions among the iron moments. There are a superexchange interaction and an indirect exchange interaction that is provided by the spin (and charge) itinerant carriers in a spin-polarized band situated in the vicinity of the Fermi level of the Fe-doped ZnO semiconductor. The former interaction actuates for an antiferromagnetic coupling among iron ions, whereas the latter constitutes a driving force for a ferromagnetic coupling that weakens, decreasing the temperature. Our results strongly contribute to the literature because they elucidate the controversies reported in the literature for the magnetic state of the Fe-doped ZnO system.

## 1. Introduction

Zinc oxide shows interesting physical and chemical properties, as well as possible technological applications [1]. One of the most important properties of ZnO is the semiconductivity at room temperature (RT), characterized by a wide bandgap (~3.37 eV) and large binding energy (60 meV) [2]. In the last two decades, ZnO doped with magnetic transition metals (i.e., TM-doped in ZnO) has received a lot of attention due to the possibility of the formation of a new class of materials, called diluted magnetic semiconductor (DMS), which could be applied in the construction of spintronic devices [3]. Indeed, the interest was triggered by the theoretical prediction of ferromagnetism at RT in TM-doped in ZnO reported by Dietl [4]. Since then, the Zn_1-x_Fe_x_O system has been prepared using several different chemical methods and has been widely characterized by its hypothetical ferromagnetic (FM) properties at RT [5]. A broad set of results, although contradictory, were reported with some authors claiming to find FM order and others refuting this property for the Fe-doped ZnO [6,7,8]. The lack of agreement may be partially explained by artifacts of sample synthesis, which eventually generate magnetic and undetectable minor magnetic phases, in addition to the Zn_1-x_Fe_x_O solid solution, and magnetic characterizations carried out without adequate precision. Therefore, the synthesis of truly monophasic Fe-doped ZnO samples remains a challenge for researchers. To ensure that the synthesized samples are single phase, the characterization techniques must be rigorously and adequately applied to identify (or not) the presence of minor magnetic phase contributions that could mask the actual magnetic state of pure oxide doped with Fe.

On the other hand, any theory on the magnetic properties of DMS should be based on knowing the consequences of TM-doping on the structure of the semiconductor energy band, which, according to our interpretation, constitutes the possible mechanism that could explain the presence of ferromagnetic ordering in DMS.

In this paper, we report the results of an investigation on the Zn_1-x_Fe_x_O system, prepared by freeze-drying an aqueous solution of zinc and iron acetate, followed by an appropriate heat treatment previously developed in our group. In fact, this method of synthesis was previously used successfully to prepare monophasic oxides doped with TM [9,10]. The Fe-doped ZnO samples were characterized by ordinary and synchrotron X-ray diffractometry (XRD), energy dispersive X-ray analysis (EDX), transmission electron microscopy (TEM), UV-VIS diffused reflectance spectroscopy, ^57^Fe Mössbauer spectroscopy, and DC and AC magnetization techniques. The results revealed the formation of monophasic Zn_1-x_Fe_x_O nanoparticles for x ≤ 0.05 and the modification of the structure of the original ZnO energy band by Fe-doping. Transitions from the paramagnetic (PM) state with existing FM correlations in RT, to a spin-glass system, were identified by lowering the sample temperature below 75 K. The frustrated magnetic state was attributed to competition between Fe atoms caused by superexchange and indirect exchange interactions. The competition mechanism between the two magnetic interactions proposed here to describe the magnetic behavior of the Zn_1-x_Fe_x_O system could explain the presence or absence of FM order in other DMS systems.

## 2. Experimental Details

Zn_1-x_Fe_x_O samples (x = 0, 0.01, 0.04, 0.05, and 0.06) were prepared by mixing the calculated quantities of Zinc(II) acetate dihydrate (Zn(CH_3_COO)_2_·2H_2_O) and Fe(III) acetate, isotopically enriched in the ^57^Fe isotope ([^57^Fe_3_O(CH_3_COO)_6_(H_2_O)_3_]CH_3_COO; see Ref. [11], for its synthesis details), which were homogeneously diluted in 50 mL distilled–deionized water. This aqueous solution was frozen inside a glass flask using liquid nitrogen, and then placed in a freeze-dryer. During the freeze-drying process, the frozen samples were sublimed under low pressure (~130 μmHg) and low temperature (−58 °C) for 24 h. The resultant lyophilized powder was heat treated in air atmosphere at 400 °C for 3 h, after using a heating rate of 10 °C/ min.

The crystalline structures were first analyzed using a conventional diffractometer (Shimadzu–XRD-6000) in the Bragg–Brentano geometry, where the data were collected in the angle range of 25 to 75° with a step of 0.02° and 2.5 s per step. So, as to precisely verify the presence of secondary phases, samples with x = 0 and 0.05 were further characterized using the XRD-1 beamline, at the LNLS (Campinas, Brazil). In this facility, the data were collected in the transmission geometry using a collimated X-ray beam. The Rietveld refinement method was applied using the free software FullProf and the crystallite size was evaluated using the Scherrer equation [12,13]. EDX analyses were performed in a scanning electron microscope (Quanta 250) and used to check the iron doping concentration. The morphologies of undoped and Fe-doped ZnO powders were studied using a transmission electron microscope (JEOL 1011). The band gap energy of the samples was obtained in a UV–VIS spectrophotometer (Perkin Elmer–LAMBDA 1050), using an integrating sphere attachment. A diffused reflectance spectroscopy measurement in the range of 200–800 nm was performed, with a resolution of 0.5 nm. The direct optical bandgap of pure ZnO and Fe-doped ZnO was estimated by diffuse reflectance spectra and analyzed by the Kubelka–Munk equation [14]. The energy of the optical band gap was determined using the Tauc plot [15].

DC magnetization *versus* temperature curves, M (T), were measured in the temperature range of 2–300 K, with a probe field of 100 Oe for all samples. Particularly for the sample x = 0.05, the curves were obtained in applied fields other than 100 Oe (see Supplementary Materials). For such measurements, the ordinary zero field cooling (ZFC) and field cooling (FC) protocols were adopted. AC magnetic susceptibility (χ′) versus temperature curves were performed in the temperature range of 10–300 K, in a 10 Oe oscillating probe field, with frequencies (f) ranging from 100 to 10 kHz. In addition, the magnetization loops versus applied magnetic field were measured at 300 and 5 K. All these measurements were made using the vibrating sample magnetometer option in a physical property measurement system (PPMS–Quantum Design - EverCool II). 

^57^Fe Mössbauer transmission spectra were taken at RT and low temperatures, in a spectrometer operating with a ^57^Co(Rh) source, moving in a constant acceleration mode. Zn_1-x_Fe_x_O powder samples measuring about 65 mg·cm^−2^ were used as absorbers. The equipment was calibrated from the spectrum of an α-Fe thin foil, also measured at RT. The low temperature measurements were run in a closed cycle He cryostat. The numerical fits were made considering a Lorentzian line shape and applying the minimum chi-square method. Each relevant and specific site was represented by a discrete subspectrum and a hyperfine magnetic field distribution was used for some low temperature spectra.

## 3. Results

### 3.1. X-ray Diffractometry

**Figure 1:**
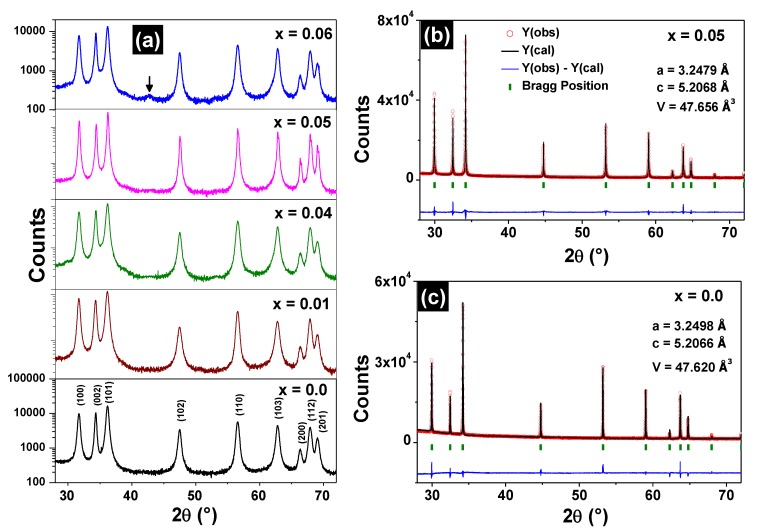
XRD patterns (ordinate in logarithmic scale) of the Zn_1−x_Fe_x_O samples (**a**). The arrow in the x = 0.06 XRD pattern indicates the (400) peak of the ZnFe_2_O_4_ phase. Refined diffractograms for the Zn_0.95_Fe_0.05_O (**b**) and ZnO (**c**) samples.

The XRD patterns of the Zn_1−x_Fe_x_O samples are shown in Figure 1a. The Bragg peaks coincide with those found for the wurtzite-like structure when x ≤ 0.05, indicating that these samples are monophasic. For x = 0.06, a secondary phase, corresponding to the ZnFe_2_O_4_ spinel, was detected in the XRD pattern. The refined parameters are listed in Table 1. It is noted that lattice parameters (*a*, *c*) and cell volumes (V) do not present any significant variation increasing the iron content. 

The refinement of the high-resolution diffractograms of the Zn_0.95_Fe_0.05_O and ZnO samples are shown respectively in Figure 1b,c and also do not indicate the presence of spurious phases. The lattice parameters obtained using synchrotron radiation experiments also match those found by conventional XRD measurements, which are shown in Table 1.

### 3.2. Chemical Analysis and Morphological Characterization

**Figure 2:**
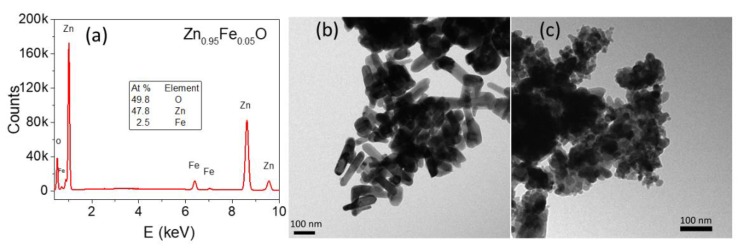
EDX spectrum of the Zn_0.95_Fe_0.05_O sample (**a**); TEM images for the ZnO (**b**) and Zn_0.95_Fe_0.05_O (**c**) samples.

Figure 2a shows the EDX spectrum for the sample Zn_0.95_Fe_0.05_O, where only oxygen, zinc, and iron peaks are identified. Therefore, this sample is free of undesirable elemental impurities, which was also verified for the other Fe-doped samples (EDX spectra not shown). The estimated iron concentrations are very close to the nominal values (see Table 1). TEM micrographs revealed nanostructured particles although with different morphologies: ZnO presents nanorod shaped particles of (5 ± 2) × 10 nm average width (Figure 2b), whereas sample Zn_0.95_Fe_0.05_O has rounded polyhedra with (29 ± 8) nm average diameter (Figure 2c).

### 3.3. Optical Characterization

**Figure 3:**
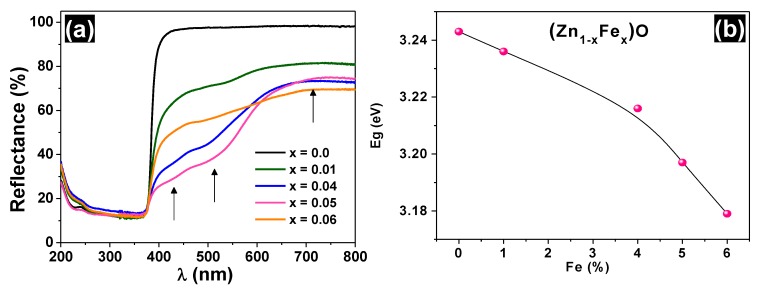
UV-Vis diffuse reflectance spectra (**a**) and bandgap variation as a function of iron concentration (**b**) for the undoped and Fe-doped ZnO.

The UV-VIS diffuse reflectance spectra of the samples are shown in Figure 3a. The reflectance profile for wavelength λ > 400 nm is significantly modified with the iron doping of ZnO. In this λ range, the line basis decreases sharply and there are visible steps of approximately 442, 516, and 728 nm (i.e., energies usually attributed to Fe^3+^ [16,17]). In addition, progressive smoothing at λ ≈ 380 nm is observed in the angular profile, increasing the iron concentration.

The band gap values of all samples are also listed in Table 1 (see also the Tauc plots in the Figure S1/Supplementary Materials). The Eg value for the ZnO sample is 3.246 eV, which is close to the value reported in the literature [17]. A decrease in the bandgap was observed as the iron content increased, as suggested in Figure 3b. This Eg behavior can be reasonably explained by the appearance of impurity bands in the original band gap and the progressive increase in the number of the respective donor or acceptor states, as discussed further.

### 3.4. Magnetic Characterization

**Figure 4:**
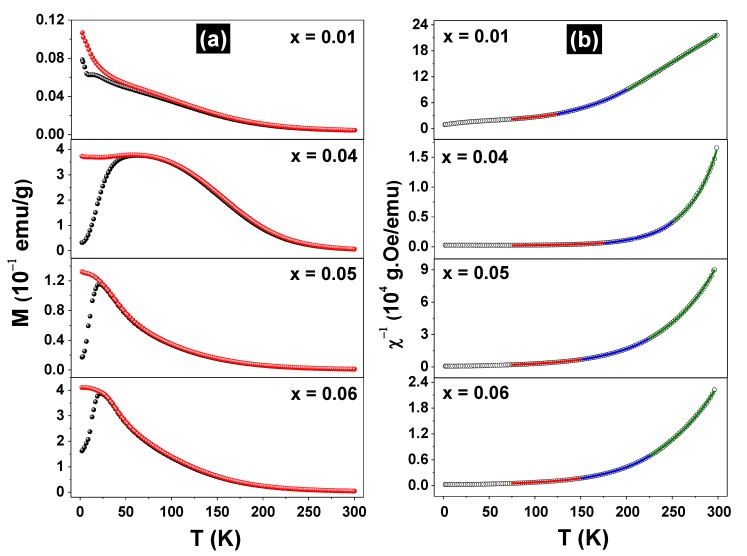
Zero field cooling (●) and field cooling (●) curves, taken with a probe field of H = 100 Oe for the (Zn_1-x_Fe_x_)O samples (**a**) and the corresponding reciprocal susceptibility fitted curves (**b**).

Figure 4a shows the ZFC and FC curves for the Fe-doped samples measured under a 100 Oe probe field.

For the sample x = 0.01, the ZFC and FC curves grew monotonically, decreasing the temperature to the lowest limit. However, a magnetic transition or spin reorientation effect in the temperature range of 6–20 K may occur in this sample. The ZFC/FC curves for the x = 0.04, 0.05, and 0.06 samples have different shapes from the previous ones, but clearly reveal magnetic transitions at 25 K (x = 0.05 and 0.06) and 50 K (x = 0.04). All pairs of ZFC and FC curves overlap largely but present irreversibility at a specific temperature that is dependent on the x value. Furthermore, it should be noted that the ZFC/FC curves for the sample x = 0.04 are quite different from the others since they extend over a larger temperature range. This feature may reflect the lack of chemical homogeneity at the nanometric scale in this sample, since small variations of the local iron concentration in the sample may spread the transition temperature in a large range. Due to the similarity with the M (T) curves obtained by others for spin-glass (SG) systems [18] and putting into consideration the distribution of the iron magnetic moments throughout the ZnO matrix, it can be inferred that the x ≥ 0.04 samples make a transition from PM to SG state at temperatures of about 25 K. To understand the magnetic behavior of the Fe-doped samples, which order only at temperatures below 75 K, we plotted the inverse of the FC susceptibility, as exhibited in Figure 4b. Obviously, these DC χ^−1^ *vs*. T curves do not reveal an ideal Curie–Weiss (C-W) behavior (i.e., a straight line), which means that the samples are composed of more than one magnetic phase and/or the magnetic correlations change along throughout the temperature range. Thus, the χ^−1^ *vs*. T curves were first adjusted using the 1/χ = 1/[χ_0_ + C/(T − θ)] equation (i.e., with a modified C-W function), taking into account the entire temperature range from 5–300 K. The independent temperature signal χ_0_ was added to the equation to account for magnetic contributions other than the specific Zn_1-x_Fe_x_O solid solution phase, which composes the sample to be characterized (e.g., the sample holder). This procedure resulted in poor adjustments between the theoretical and experimental 1/χ curves. Other possibilities were also tested, such as dividing the temperature range into two or three segments and applying the above equation again. It resulted in adjustments considered satisfactory only for high temperatures segments, say T > 200 K. Good adjustments were finally obtained using the 1/χ = 1/[χ_0_ + C/(T − θ)^n^] equation and taking into account separately the 225 K ≤ T ≤ 300 K, 150 K ≤ T ≤ 225 K, and 75 K ≤ T ≤ 150 K temperature ranges (ΔT′s), with n = 1, 1.1, and 1.2 for each range, respectively. The lower temperature limit was chosen because all samples have frozen iron moments near or below 75 K, as confirmed by Mössbauer data (see below). The adjusted parameters are listed in Table 2.

For the higher and intermediate temperature ranges, all C-W coefficients (θ′s) are positive or slightly negative (only for the x = 0.01 sample), whereas they are negative for the lowest temperature range. The trend is such that θ′s decrease with the temperature range, in correlation with n for all samples. The positive coefficients reflect FM correlations between the iron moments, although in all cases the interactions between the magnetic moments are weak and are overcome by thermal fluctuations, even at intermediate temperatures. Furthermore, their tendency to become negative at low temperatures is consistent with the transition model to an SG state [18]. θ′s are similar for the x = 0.05 and 0.06 samples, but they differ from those of the x = 0.04 sample, an effect that may be reflecting the largest peak observed in Figure 4a. The x = 0.01 sample shows the smallest θ′s, which indicates that the transition PM → SG occurs at much lower temperatures for this sample. It is worthy to note that n > 1 has been reported for SG systems [19]. 

On the other hand, the parameters C′s also correlate consistently with n, though they increase when the temperature range decreases. This plausibly means that the iron magnetic moment or its effective value, ***μ_Fe_***, increases reducing the temperature, as C ~ ***μ_Fe_*** [20]). The small and negative χ_0_ values are typical of diamagnetic components but also change with ΔT and x, showing more than a simple diamagnetic signal.

The FC curves for the Zn_0.95_Fe_0.05_O sample, taken with different applied magnetic fields, can be seen in the Figure S2 (Supplementary Materials).

**Figure 5:**
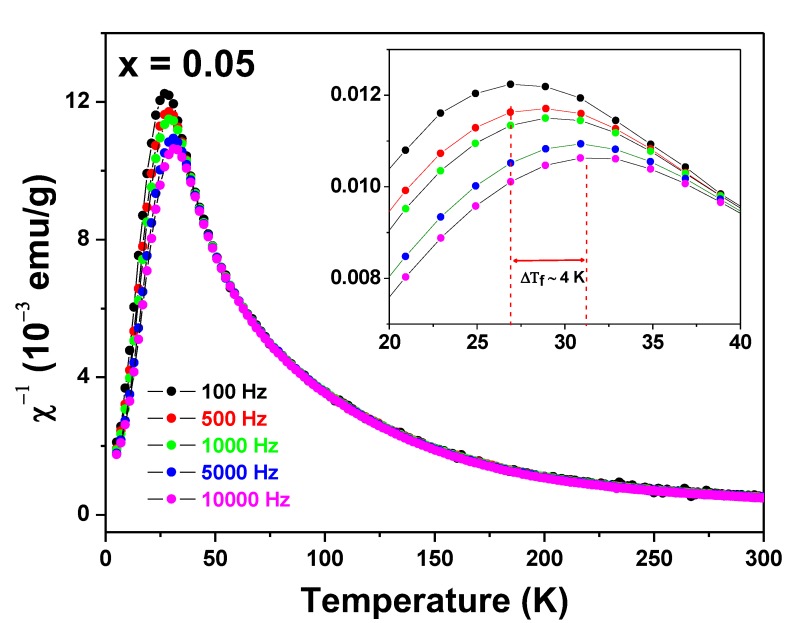
AC magnetic susceptibility χ′(Τ, f) curves of the Zn_0.95_Fe_0.05_O sample. The frequencies are shown in the figure. The insert displays the peak shifting effect due to the frequency change of the experiment.

The interpretation of a PM → SG transition, was further confirmed by AC magnetic susceptibility *versus* temperature measurements taken for the Zn_0.95_Fe_0.05_O sample, scanning at different frequencies, as shown in Figure 5. The χ′(T, f) curves taken for the Zn_0.95_Fe_0.05_O sample are the signature of a canonical spin-glass system [21,22].

The sharp peak associated with the spin freezing temperature shifts about 4 K (=ΔT_f_) to higher temperatures when the frequency of the probe field increases (Δf ~ 10 kHz). Interestingly, these χ′(T, f) curves are similar to those obtained by us for a Zn_0.95_Fe_0.05_O sample prepared by a different synthesis method [23].

**Figure 6:**
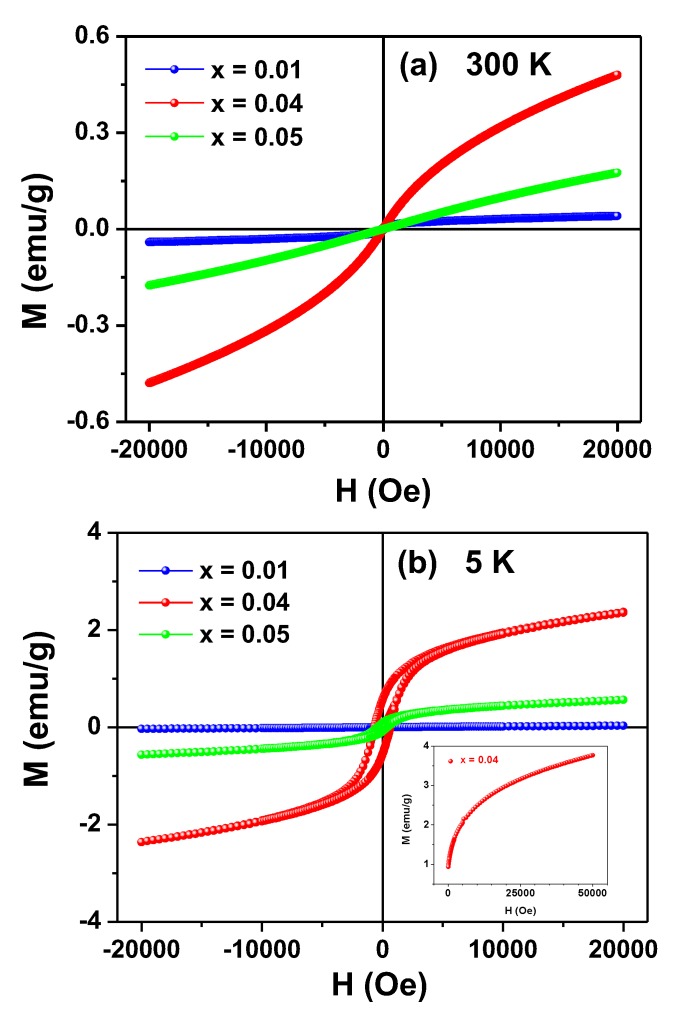
M (H) curves taken at 300 K (**a**) and 5 K (**b**) for the Zn_1-x_Fe_x_O samples. The insert in (**b**) shows the initial magnetization curve for the x = 0.04 sample up to the limit field of 50 kOe (μ_o_H = 5 T).

Magnetization *versus* applied magnetic field curves obtained at 300 and 5 K for the Zn_1-x_Fe_x_O samples are shown in Figure 6. 

None of the M (H) curves reached saturation under applied fields of up to 20 kOe. In fact, the sample x = 0.04 did not reach saturation even for H = 60 kOe at 5 K (see insert in Figure 6b), exposing another evidence of the SG state of the samples. 

The curves measured at RT (Figure 6a) are typical for PM systems, although a faint hysteretic contribution can be identified by closely inspecting the curve of the sample x = 0.01. In a different way, the M (H) curves obtained at 5 K for the x ≥ 0.04 samples showed clear magnetic irreversibility, whereas for the x = 0.01 sample only a modest hysteresis could be observed by a zoom (not shown) in the region of low applied fields.

### 3.5. Mössbauer Spectroscopy

**Figure 7:**
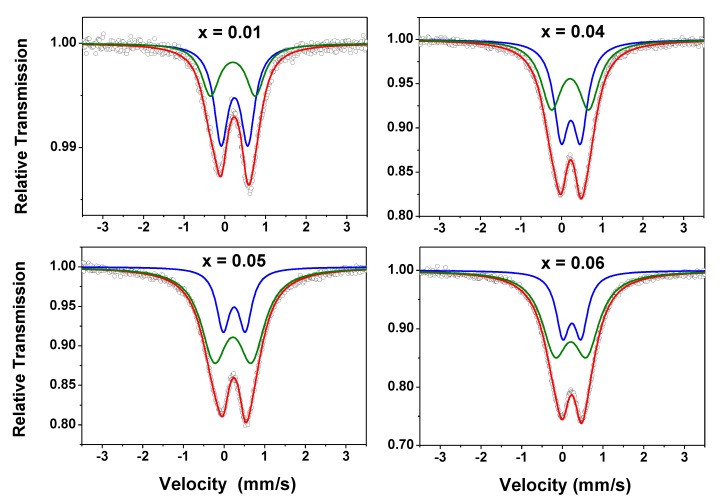
RT Mössbauer spectra taken with |Vmax| = 3.5 mm/s for the Zn_1-x_Fe_x_O samples. The two subspectra (green and blue lines) used to fit the data are also shown in this figure.

RT Mössbauer spectra of the Zn_1-x_Fe_x_O samples are shown in Figure 7. A preliminary analysis did not reveal any magnetic components. This is consistent with the magnetization results (i.e., no magnetic order at RT), except for the x = 0.01 sample, which showed a slight irreversibility in the M (H) curve.

The spectra here obtained are similar to those reported by other authors for iron diluted in zinc oxide [24] and by us for Zn_1-x-y_Fe_x_Co_y_O samples [23]. They were fitted with two doublets (Doublet I and Doublet II), i.e., only paramagnetic components, and the hyperfine parameters are listed in Table 3.

Analyzing the evolution with x of the subspectral areas—which reveals a tendency to increase Doublet II at the expense of Doublet I—it is plausible to attribute Doublet I to isolated iron atoms and Doublet II to iron atoms with one or more neighboring iron atoms (in every case, iron substituting zinc in the distorted 2b site in the wurtzite lattice). This interpretation is also corroborated by our study on the Zn_1-x-y_Fe_x_Co_y_O system [23].

The quadrupole splitting (ΔE_Q_) values are comparable to the average values reported in the literature [24], where the authors also considered two doublets for fitting their spectra of (Zn, Fe)O solid solutions. Based on the values of the isomer shifts (δ), it follows that iron is in the trivalent state (high-spin), although the ΔE_Q_ of Doublet II is unusually high for the ferric cation. This high ΔE_Q_ value can be attributed, in addition to the non-cubicity of the crystallographic site, to a lattice distortion caused by the replacement of zinc by iron. Due to the difference between the valence states of Fe^3+^ and Zn^2+^ ions, it is expected that the cation replacement generates zinc vacancies in the wurtzite matrix, further increasing the lattice distortion. It is reasonable to assume that for each pair of substitutional iron atoms, a zinc vacancy is created to preserve the local electroneutrality in the lattice. In fact, we assume that the substitutional solute atom-vacancy complex, consisting of two iron atoms (as suggested above) plus a zinc vacancy, all separated by one bond length, is a predominant configuration, resulting from the iron doping process.

As suggested by χ′(T, f) data, Fe atoms have magnetic moment and the fact that Zn_1-x_Fe_x_O solid solutions showed no clear magnetic hyperfine pattern at RT indicates that the iron magnetic moments relax faster than the Mössbauer spectroscopy time window. As stated earlier, these results are consistent with the above magnetization analyses, which revealed a PM state for the Zn_1-x_Fe_x_O system, despite observable FM correlations at RT.

**Figure 8:**
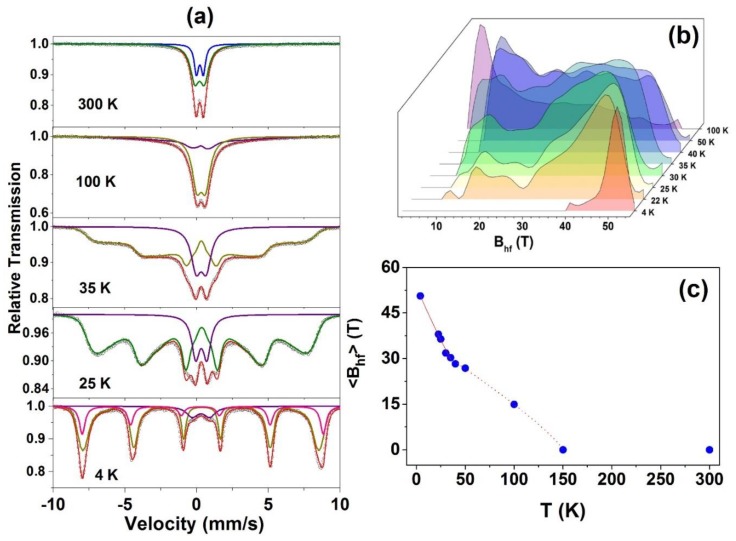
Room temperature and low temperature Mössbauer spectra taken with |Vmax| = 10 mm/s for the Zn_0.95_Fe_0.05_O (**a**), all the Bhf distributions (**b**), and <Bhf> *versus* temperature for this sample (**c**).

Some Mössbauer spectra of the Zn_0.95_Fe_0.05_O sample obtained at temperatures below RT are shown in Figure 8. Their hyperfine parameters are also listed in Table 3. The partially split magnetic spectra obtained at temperatures well below RT were fitted using a doublet (designated herein by Doublet III) and a hyperfine magnetic field distribution (B_hf_ Dist.). The Doublet III is the superposition of both Doublets (I and II) that could not be unambiguously resolved in the fits with B_hf_ distribution, i.e., it has hyperfine parameters that are average values between those of Doublet I and Doublet II (see Table 3). 

On the other hand, the analysis of the spectral evolution of all samples under decreasing temperature showed that as a general trend, the higher the x, the higher the temperature to begin the magnetic split of the spectrum. Thus, it can be inferred that this initial magnetic fraction of the spectra can be attributed to clusters of two or more neighboring iron atoms, which previously contributed to Doublet II. Although the Doublet II is supposed to be the first to convert and reveal a magnetic character (assumption that can be explained considering this component as coming from iron clusters), eventually the magnetic fraction of any spectrum increases at the expense of the PM fraction, formerly contributing to the Doublet I. This means that the isolated Fe magnetic moments also experience a slowdown relaxation and spin freezing effects as the sample temperature decreases.

For the lowest temperature measurement (4 K) taken for the x = 0.05 sample, a discrete sextet was added to the fit to account for some asymmetry in the B_hf_ Dist. spectral fraction. The new magnetic component presents a quadrupole interaction that is different from that of the B_hf_ Dist. This likely reflects a fraction of the iron atoms with a proper crystallographic configuration, eventually those with several neighboring ferric cations. Finally, there is a notable increase in the ∆E_Q_ value of the Doublet III when the temperature decreases, revealing a significant cubic distortion of the cationic site in the wurtzite structure at very low temperatures. In fact, this is an expected temperature effect for ∆E_Q_. All this spectral behavior under temperature variation is consistent with a PM → SG magnetic transition, as already indicated by the magnetization curves shown in Figure 4 and Figure 5. In other words, magnetic splitting only starts at low temperatures and is also dependent on the value of x (see the low-temperature spectra for x = 0.01 and 0.04 samples, in Figure S3/Supplementary Materials).

The temperature evolution of B_hf_ shown in Figure 8c for x = 0.05 sample reinforces the PM → SG interpretation since it reproduces qualitatively the analogous B_hf_ behavior found in the literature for well-known SG systems [25,26]. 

## 4. Discussion

**Figure 9:**
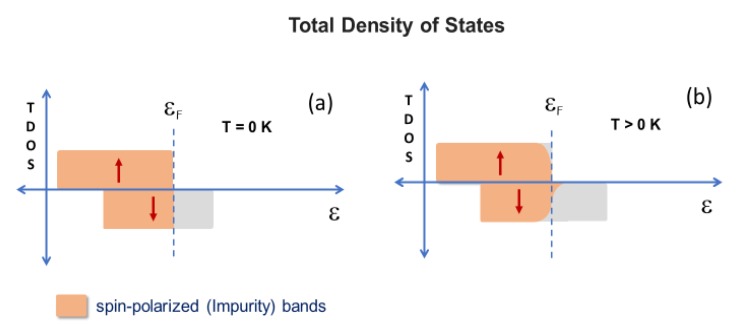
Total density of states: scheme proposed for the doped semiconductor at T = 0 K (**a**) and T > 0 K (**b**).

The change in the magnetic behavior of the samples studied under temperature variation can be interpreted based on the simultaneous occurrence of two types of magnetic exchange interactions between the Fe impurities diluted in the wurtzite-like structure. Both may depend on the concentration of iron or temperature and are mutually competitive. One is the superexchange interaction, which involves two neighboring iron moments (i.e., Fe^3+^ cations as shown by Mössbauer spectroscopy), mediated by an oxygen anion. It is more significant when the concentration of iron moments in the ZnO matrix is higher since the pairs of adjacent magnetic moments are more numerous for higher x. Depending on the configuration of two neighboring ferric cations, the coupling is parallel or antiparallel, as established by several first principle calculations [27,28]. However, in general, these theoretical studies do not consider the presence of cationic vacancies close to the localized moments. Certainly, taking into account this complex vacancy-dopant defect would give simulations much more sophisticated, but more realistic magnetic energy values would be achieved. Here, we assume that the antiparallel coupling is the most stable, as indicated by some numerical studies. At least for x = 0.04 and 0.05, the interaction would percolate the crystal, leading to a long-range AFM order in the absence of thermal agitation or any other competitive interaction. Indeed, the magnetic frustration (i.e., the spin-glass state) observed for samples at low temperatures indicates that there is a magnetic competition. Plausibly, it comes from the indirect exchange interaction between iron moments, mediated by charge (and spin) carriers (i.e., electrons and/or holes) in the impurity bands created by the iron doping of the ZnO matrix. Impurity bands are created because Fe^3+^ cations are not isovalent to Zn^2+^ and may introduce cationic vacancies in the matrix, as discussed in the Mössbauer section. The iron cations may trap electrons in hydrogenic orbits, while vacancies have holes available to trap. Each iron cation/zinc vacancy exerts a proper central force under which the carrier charge (i.e., the electron/the hole) is bound. When electrons/hole orbitals overlap spatially, extended donor/acceptor levels are formed, and the density of states depends on the concentration of iron. Presumably, these are shallow orbitals, i.e., the acceptor levels are just above the top of the valence band, whereas donor levels are right below the bottom of the conduction band. The number of donor and acceptor levels is the same since a vacancy is created for each pair of dopants. Therefore, magnetic cations and vacancies that share the same region in the ZnO matrix form two impurity bands inside the gap. However, the band structure of the semiconductor Zn_1-x_Fe_x_O may be more complex due to the partial degeneration of the Fe^3+^ d states (i.e., t_2g_ and e_g_ orbitals) due to the crystalline field. Thus, in the absence of a theoretical calculation and considering all the factors that could determine the Zn_1-x_Fe_x_O energy band structure, we propose a simple description of the total density of states (TDOS), shown in Figure 9.

A spin-polarized band (say, spin down) is situated around the Fermi level. The itinerant spin (and charge) carriers occupying this band would be responsible for an indirect exchange interaction, inducing FM coupling between localized iron moments. As considered above for AFM coupling, this interaction could lead to a long-range FM order in the absence of thermal agitation or any other competitive interaction. For a given doping concentration, the occupation of the polarized band increases at “higher” temperatures, which increases the ability to ferromagnetically order the localized iron moments. The same augmentation could be achieved at some fixed temperature, simply by increasing the concentration of doping. In the present case, it was observed that either the thermal fluctuations or the competitive AFM interaction between iron magnetic moments prevented the FM ordering and all the systems were unordered at RT. However, the occurrence of FM correlations when fitting the 1/χ curves indicates that the indirect interaction overcomes the direct (superexchange) interaction at temperatures close or just below RT, although it is not strong enough to induce an effective FM order. As the temperature decreases, the occupation of this band decreases, weakening the indirect interaction between the iron moments or their parallel coupling. While crossing down some temperature, when thermal fluctuations are not significant, both competing magnetic interactions tend to equalize, resulting in the spin-glass states observed for the Fe-doped system.

In summary, quantitative theoretical models should be developed to determine which semiconductor-dopant pairs and the adequate doping concentration for which the energy band structure generates an FM coupling between the localized magnetic moments strong enough to overcome the competition with the AFM coupling between them and the thermal energy at RT.

## 5. Conclusions

- The process of lyophilization of the aqueous solution of iron and zinc acetates, followed by an appropriate heat treatment of the lyophilized material, resulted in Zn_1-x_Fe_x_O nanoparticles, which are monophasic for x ≤ 0.05;
- Applying this synthesis routine, Fe-doped ZnO induced a morphological change in the crystallites, from the ZnO nanorods to the Zn_0.95_Fe_0.05_O round polyhedral;
- The iron cations are trivalent and substitutional for zinc in the wurtzite matrix and can be divided into two groups: isolated iron atoms and those with one or more neighboring iron atoms.
- The band gap of the semiconductor decreases while a spin-polarized band situated close to the Fermi level emerges in the semiconductor band structure, with iron doping;
- The x ≤ 0.05 Zn_1-x_Fe_x_O samples have no magnetic order at RT, but undergo a transition from the PM state to an SG-like state as the sample temperature decreases; this magnetic transition passed through, at least, three different magnetic regimes.
- The magnetic frustration of localized iron moments at low temperatures is a result of the competition between indirect and direct superexchange interactions between the localized iron moments.

## Figures and Tables

**Table 1:** Crystallographic parameters, refinement goodness (S = R_wp_/R_exp_), crystallite sizes (D), measured iron concentrations (x_exp_), and energy band gaps obtained for the Zn_1−x_Fe_x_O samples.

Sample	a (Ȧ)	c (Ȧ)	V (Ȧ^3^)	S	D (nm)	*x_exp_* (%)	E_g_ (eV)
ZnO	3.2499(2)	5.2067(3)	47.63 (5)	1.86	40(2)	-	3.243(2)
Zn_0.99_Fe_0.01_O	3.2486(1)	5.2101(1)	47.62 (1)	2.44	25(1)	0.90	3.236(1)
Zn_0.96_Fe_0.04_O	3.2465(1)	5.2054(2)	47.51 (1)	2.17	25(1)	3.74	3.216(1)
Zn_0.95_Fe_0.05_O	3.2479(1)	5.2012(3)	47.51 (1)	2.37	45(2)	4.97	3.197(1)
Zn_0.94_Fe_0.06_O	3.2482(1)	5.2057(2)	47.57 (1)	1.88	26(2)	5.67	3.195(1)

**Table 2:** Parameters obtained by fitting the χ^−1^(T) curves taken with H = 100 Oe for the (Zn_1-x_Fe_x_)O samples.

Sample (x)	ΔT	θ (K)	C (K.emu/g.Oe)	χ_0_ (10^−5^ emu/g.Oe)	n
0.06	225–300 K	156	0.014	−5.36	1
	150–225 K	77	0.096	−24.60	1.1
	75–150 K	−53	1.078	−125.00	1.2
0.05	225–300 K	150	0.004	−1.73	1
	150–225 K	71	0.026	−6.53	1.1
	75–150 K	−37	0.216	−25.90	1.2
0.04	225–300 K	193	0.022	−15.10	1
	150–225 K	114	0.310	−23.20	1.1
	75–150 K	−168	1.720	−29.00	1.2
0.01	225–300 K	123	0.009	−1.83	1
	150–225 K	−12	0.011	−20.00	1.1
	75–150 K	−770	0.820	−0.06	1.2

**Table 3:** Hyperfine parameters and subspectral areas for the Zn_1-x_Fe_x_O samples recorded at different temperatures.

Sample (x)	T(K)	Site	δ (mm/s) * (± 0.01)	∆E_Q_/2ε (mm/s) (± 0.02)	Bhf (T) ** (± 0.5)	Γ (mm/s) (± 0.02)	Area (%) (± 1)
0.01	300	Doublet I	0.35	0.66	–	0.42	57
		Doublet II	0.31	1.10	–	0.53	43
	50	B_hf_ Dist.	0.48	−0.07	26.1	–	43
		Doublet III	0.47	0.80	–	0.75	57
	25	B_hf_ Dist.	0.50	−0.07	39.3	–	58
		Doublet III	0.47	0.76	–	0.96	42
0.04	300	Doublet I	0.34	0.48	–	0.43	50
		Doublet II	0.32	0.92	–	0.61	50
	50	B_hf_ Dist.	0.43	−0.02	29.4	–	50
		Doublet III	0.44	0.74	–	0.99	50
	25	B_hf_ Dist.	0.45	−0.02	37.7	–	74
		Doublet III	0.42	0.79	–	0.68	26
0.05	300	Doublet I	0.36	0.55	–	0.40	26
		Doublet II	0.33	0.92	–	0.79	74
	150	Doublet I	0.36	0.55	–	0.33	25
		Doublet II	0.32	0.87	–	0.76	75
	100	B_hf_ Dist.	0.40	−0.08	14.9	–	35
		Doublet III	0.44	0.61	–	0.78	65
	50	B_hf_ Dist.	0.43	−0.02	26.6	–	52
		Doublet III	0.44	0.67	–	0.96	48
	40	B_hf_ Dist.	0.43	−0.01	28.3	–	71
		Doublet III	0.44	0.69	–	0.69	29
	35	B_hf_ Dist.	0.43	−0.02	30.3	–	80
		Doublet III	0.43	0.70	–	0.83	20
	30	B_hf_ Dist.	0.43	−0.07	31.8	–	86
		Doublet III	0.42	0.64	–	0.64	14
	25	B_hf_ Dist.	0.45	−0.02	36.4	–	87
		Doublet III	0.43	0.78	–	0.78	13
	22	B_hf_ Dist.	0.45	−0.07	38	–	88
		Doublet III	0.42	0.78	–	0.77	12
	4.2	Sextet	0.46	0.17	52.1	0.47	25
		B_hf_ Dist.	0.45	−0.08	50.0	*	65
		Doublet III	0.42	1.22	–	0.96	10
0.06	300	Doublet I	0.35	0.44	–	0.39	30
		Doublet II	0.32	0.77	–	0.73	70

δ = isomer shift; ∆E_Q_/2ε = quadrupole splitting/idem, in the presence of magnetic interaction; B_hf_ = magnetic hyperfine field; Γ = linewidth; * relative to α-Fe at RT; ** average value in case of B_hf_ Dist.

## Supplementary Materials

The following are available online at https://www.mdpi.com/1996-1944/13/4/869/s1, Figure S1: Tauc plots for the undoped and Fe-doped ZnO. Figure S2: FC curves for the Zn_0.95_Fe_0.05_O sample, taken with different applied magnetic fields. For the highest fields and at the lowest temperatures, the frustration is removed. Figure S3: Mössbauer spectra taken with |Vmax| = 10 mm/s for the Zn_0.99_Fe_0.01_O sample at RT (a), 50 K (b) and 25 K (c) and for the Zn_0.96_Fe_0.04_O sample at RT (d), 50 K (e) and 25 K (f). The inserts represent the hyperfine magnetic field distributions.
