# Supplementary material for: Spin-Glass Transitions in Zn1-xFexO Nanoparticles
Source: Materials (Basel). 2020 Feb 14;13(4):869. doi: 10.3390/ma13040869 (PMC7079620; doi:10.3390/ma13040869)
Supplement: Supplementary file 1 [file materials-13-00869-s001.pdf]

materials

MDPI

Supplementary Materials

Spin-Glass Transitions in $Zn_{1-x}Fe_xO$ Nanoparticles

Lilian Felipe S. Tupan [1], Marlon I. Valerio-Cuadros [1], Aline Alves Oliveira [1], Reginaldo Barco [1], Flávio Francisco Ivashita [1], Lutiene F. Lopes [2], Edson C. Passamani [3] and Andrea Paesano Jr. [1,*]

[1] Departamento de Física, Maringá State University, Av. Colombo, 5790 – Zona 7, 87020-900 Maringá, PR, Brazil; lilian.tupan@hotmail.com (L.F.S.T.); marlon190@gmail.com (M.I.V.C); alineuem@hotmail.com (A.A.O.); r_barco@hotmail.com (R.B.); fivashita@gmail.com (F.F.I)

[2] Instituto de Física, Federal University of Rio Grande do Sul, 91501-970 Porto Alegre, RS, Brazil; lutienefl@gmail.com

[3] Departamento de Física, Federal University of Espírito Santo, 29075-010 Vitória, ES, Brazil; edson@cce.ufes.br

* Correspondence; apjunior@uem.br; Tel.: 55-44-3011-5928;

Received: 15 December 2019; Accepted: 6 January 2020; Published: 14 February 2020

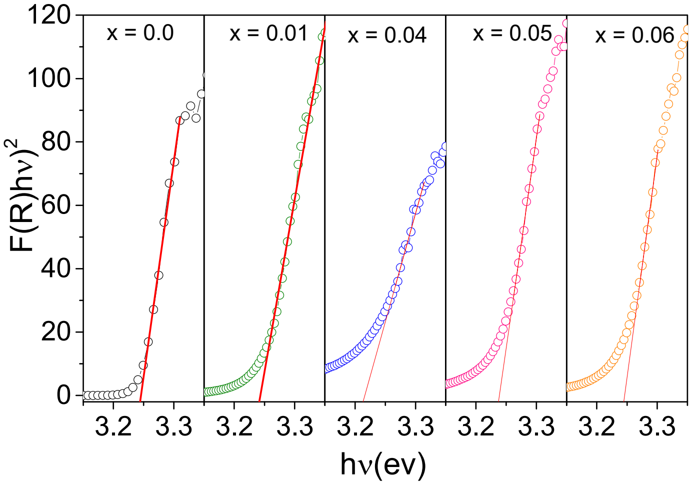

Figure S1. Tauc plots for the undoped and Fe-doped ZnO.

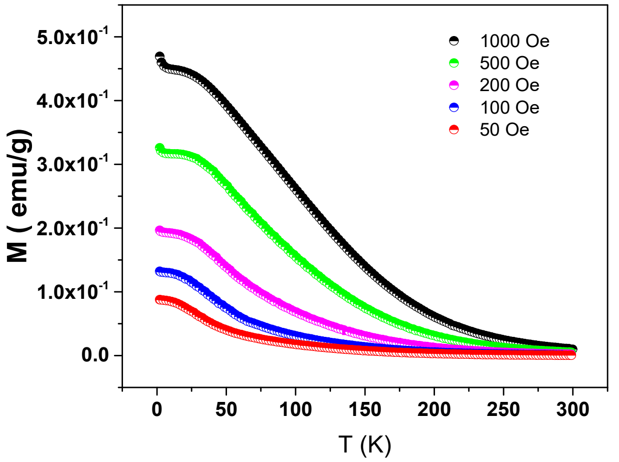

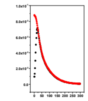

Figure S2. FC curves for the $Zn_{0.95}Fe_{0.05}O$ sample, taken with different applied magnetic fields. For the highest fields and at the lowest temperatures, the frustration is removed.

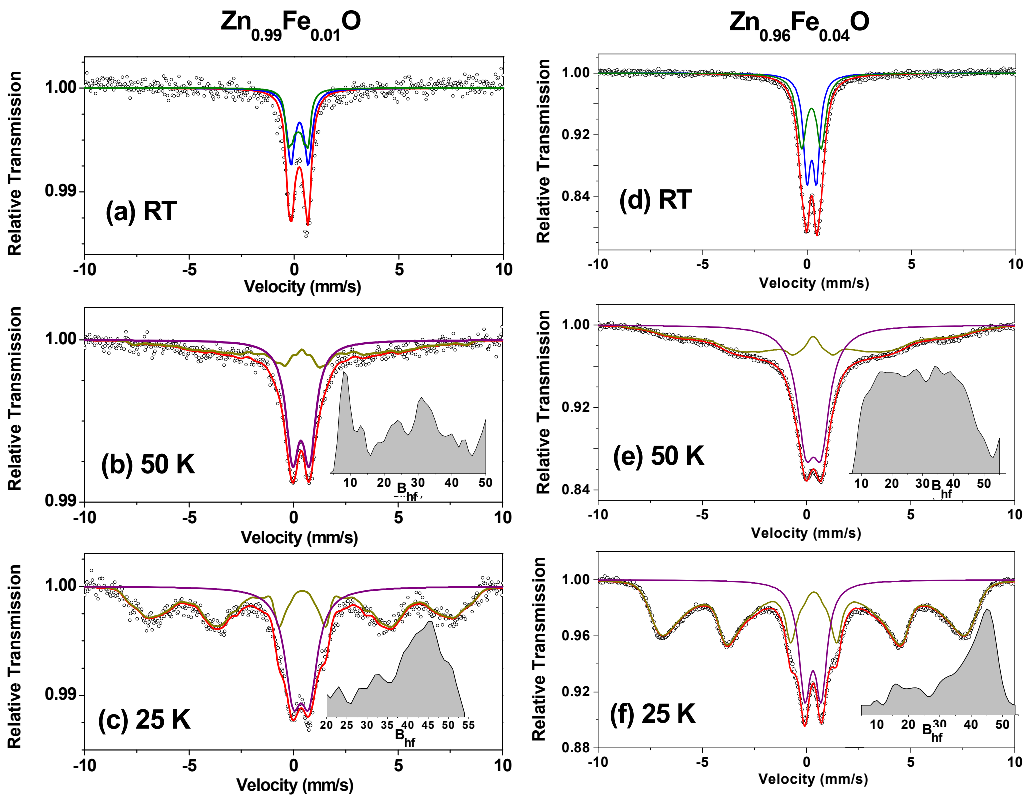

Figure S3. Mössbauer spectra taken with |Vmax| = 10 mm/s for the $Zn_{0.99}Fe_{0.01}O$ sample at RT (**a**), 50 K (**b**) and 25 K (**c**) and for the $Zn_{0.96}Fe_{0.04}O$ sample at RT (**d**), 50 K (**e**) and 25 K (**f**). The inserts represent the hyperfine magnetic field distributions.

© 2020 by the authors. Licensee MDPI, Basel, Switzerland. This article is an open access article distributed under the terms and conditions of the Creative Commons Attribution (CC BY) license (http://creativecommons.org/licenses/by/4.0/).
